# Unusual finding of endocervical-like mucinous epithelium in continuity with urothelium in endocervicosis of the urinary bladder

**DOI:** 10.1186/1746-1596-6-56

**Published:** 2011-06-23

**Authors:** Phaik-Leng Cheah, Lai-Meng Looi, George Eng-Geap Lee, Kean-Hooi Teoh, Kein-Seong Mun, Abdul Rahman Nazarina

**Affiliations:** 1 Department of Pathology, Faculty of Medicine, University of Malaya, 50603 Kuala Lumpur, Malaysia; 2 Department of Surgery, Faculty of Medicine, University of Malaya, 50603 Kuala Lumpur, Malaysia

## Abstract

Endocervicosis in the urinary bladder is a rare benign condition. We present a case in a 37-year-old woman with classical clinical and pathological features of endocervicosis. The unusual observation of endocervical-like mucinous epithelium in continuity with the urothelium in addition to fully developed endocervicosis prompted immunohistochemical profiling of the case using antibodies to cytokeratins (AE1/AE3, CK19, CK7, CK5/6, CK20), HBME-1, estrogen receptor (ER) and progesterone receptor (PR) to assess the relationship of the surface mucinous and endocervicosis glandular epithelia. The surface mucinous epithelium, urothelium and endocervicosis glands were immunopositive for AE1/AE3, CK7 and CK19 while CK20 was only expressed by few urothelial umbrella cells. The surface mucinous epithelium was CK5/6 and HBME-1 immunonegative but showed presence of ER and PR. This was in contrast to the urothelium's expression of CK5/6 but not ER and PR. In comparison, endocervicosis glands expressed HBME-1, unlike the surface mucinous epithelium. The endocervicosis epithelium also demonstrated the expected presence of ER and PR and CK5/6 immunonegativity. The slightly differing immunohistochemical phenotypes of the surface mucinous and morphologically similar endocervicosis glandular epithelium is interesting and requires further clarification to its actual nature. The patient has remained well and without evidence of disease 18-months following transurethral resection of the lesion.

## Background

Endocervicosis in the urinary bladder is a rare benign condition, first recognised by Steele and Byrne in 1982 in their report of endocervical-like glands deep in the urinary bladder wall [1]. This lesion was identified as a distinct entity by Clement and Young in 1992 [2] and the glands subsequently noted to be similar to endocervical glands in their immunohistochemical expressions [3-5]. To the best of our knowledge, there are to date less than 40 cases reported in the world literature in the two decades since this entity was first described. Usually occurring in women of reproductive age and located in the posterior bladder wall, endocervicosis is generally thought to be an embryological disorder of the secondary mullerian system [6,7] and the mucinous analogue of mullerianosis; "mullerianosis" being a term first used by Young and Clement to encompass endocervicosis, endometriosis and endosalpingiosis in the bladder [6]. Implant following pelvic surgery has also been considered an aetiological possibility as some cases were associated with earlier pelvic surgery [4,8] while others [8-10] put forth metaplasia as another possible cause of this condition. Nonetheless, the aetiogenesis of this interesting lesion still remains an enigma and largely based on circumstantial evidence. We present a case where mucinous epithelium, morphologically similar to endocervical epithelium, was detected in continuity with urothelium in addition to the characteristic endocervicosis glands, a finding that has hitherto not been reported, although Young and Clement had noted tubal epithelium replacing urothelium in mullerianosis [6]. The surface mucinous epithelium, its adjacent urothelium and the endocervicosis glands were compared for their immunohistochemical expressions of cytokeratins (AE1/AE3, CK19, CK7, CK5/6, CK20), HBME-1, estrogen receptor (ER) and progesterone receptor (PR) to assess their possible associations and provide any further insight into the pathogenesis of this rare but nevertheless clinically significant entity as well as to record the first Malaysian case.

## Case Presentation

A 37-year-old woman presented with complaint of an episode of macroscopic, painless hematuria. She had a similar episode 3-months previously and was seen at another hospital and told to have a small bladder lesion detected by ultrasonography. There was no history of catamenial exacerbation or association for either episode. She defaulted follow-up till this current episode. Apart from laparoscopic removal of an ovarian cyst (5 × 4 × 2 cm) six years ago at a different hospital, the patient was otherwise well. Review of the hematoxylin and eosin stained sections confirmed that the ovarian cyst was a benign mucinous cystadenoma lined by single-layered endocervical-like epithelium. The patient also gave a history of two caesarean sections, thirteen and eleven years earlier. Physical examination revealed no significant findings. The patient underwent cystoscopic examination and transurethral resection of the lesion at the posterior dome of the bladder under spinal anesthesia.

**Figure 1:**
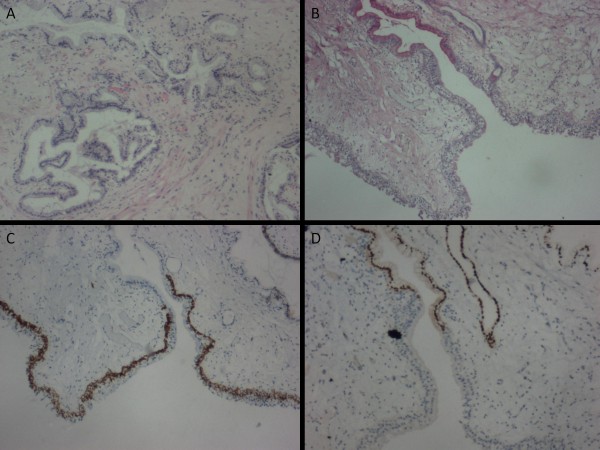
**Histologic findings of the transurethral resected bladder tissue**. (A) Endocervicosis showing round to branched endocervical-like glands with surrounding smooth muscle in the bladder (H + E × 40). (B) Surface mucinous epithelium delineated from urothelium (Southgate's mucicarmine x 40). (C) CK5/6 decorating basal cells of urothelium while surface mucinous epithelium is immunonegative (x 40). (D) Nuclear ER immunopositivity is noted in the surface mucinous epithelium and adjacent endocervicosis gland (x 40).

Multiple rubbery, whitish-grey tissue fragments of varying sizes and shapes, measuring 1.5 × 1.2 × 0.5 cm in aggregate were resected. The fragments of bladder tissue revealed glands lined by a single-layered mucinous columnar epithelium with basal nuclei reminiscent of endocervical epithelium in the lamina propia and extending into the muscularis propia. These endocervicosis glands ranged from round to branched (Figure 1A). Occasional glands were cystically dilated and contained mucin. The overlying urothelium was intact and generally unexceptional except for alteration to single-layered mucinous columnar epithelium that resembled endocervical epithelium in a few areas. Mitotic activity was not detected in any of the tissue components. The lamina propia was mildly edematous with focal congestion of the vasculature and infiltrate of lymphocytes, plasma cells, neutrophils and eosinophils. Rare hemosiderin-laden macrophages were testimony to hemorrhagic episodes.

4-μm sections of the formalin-fixed, paraffin-embedded tissue were subjected to immunohistochemical staining using monoclonal antibodies to pancytokeratin AE1/AE3 (DakoCytomation AE1/AE3, 1:100), CK7 (DakoCytomation OV-TL 12/30, 1:100), CK19 (DakoCytomation RCK108, 1:100), CK5/6 (DakoCytomation D5/16 B4, 1:100), CK20 (DakoCytomation K_s_20.8, 1:100), HBME-1 (DakoCytomation HBME-1, 1:100), estrogen receptor (ER) (NeoMarkers SP1, 1:100) and progesterone receptor (PR) (DakoCytomation PgR 636, 1:200) via the EnVision™^+ ^(DakoCytomation) system.

Southgate's mucicarmine clearly delineated the mucinous epithelium from the adjacent urothelium in this case (Figure 1B). Table 1 summarises the immunohistochemical expression profile of the urothelium, surface mucinous epithelium and endocervicosis glands. As expected, AE1/AE3, CK7 and CK19 were ubiquitously expressed by all epithelial components. CK 5/6 was noted in the basal cells of the urothelium while the surface mucinous and endocervicosis epithelia were immunonegative (Figure 1C). Faint CK20 expression was only noted in few umbrella cells of the urothelium. HBME-1 was expressed on the apical margin of endocervicosis glands. In contrast, the surface mucinous epithelium and urothelium were immunonegative. The endocervicosis glandular and surface mucinous epithelial cells demonstrated nuclear ER (Figure 1D). Endocervicosis glandular cells generally demonstrated nuclear PR with rare cells exhibiting mixed cytoplasmic and nuclear positivity. Contrastingly, PR was expressed in both the nuclei and cytoplasm of the surface mucinous epithelial cells while no PR was seen in the urothelium.

**Table 1:** Antibody expressions in urothelium, surface mucinous epithelium and endocervicosis glands

Antibody	Urothelium	Surface mucinous epithelium	Endocervicosis glands
**AE1/AE3**	Positive	Positive	Positive
**CK7**	Positive	Positive	Positive
**CK19**	Positive	Positive	Positive
**CK5/6**	Positive (basal cells)	Negative	Negative
**CK20**	Positive (umbrella cells)	Negative	Negative
**HBME-1**	Negative	Negative	Positive
**ER**	Negative	Positive	Positive
**PR**	Negative	Nuclear and cytoplasmic positivity	Nuclear positivity

## Discussion

Endocervicosis in the urinary bladder is an uncommon benign entity but causes sufficient clinical anxiety with presentation as a lesion in the bladder frequently associated with hematuria. To the best of our knowledge, there are less than 40 cases reported in the English literature to date. Table 2 summarises the clinical presentation of documented cases of bladder lesions with presence of endocervical glandular tissue [1-23]. Our case, a 37-year-old woman who presented with hematuria and a lesion in the posterior dome of the bladder together with a past history of previous caesarean sections and ovarian cystectomy has an almost classical presentation of endocervicosis. The histological features as well as the immunohistochemical profile of the endocervicosis glands were also typical. This case is however interesting in that for the first time, mucinous epithelium, morphologically similar to endocervical glandular epithelium, was observed in continuity with surface urothelium apart from the characteristic endocervicosis glands. Nevertheless, the slightly variant immunohistochemical expressions of the surface mucinous and endocervicosis epithelium are notable. Although it exhibited ER and PR immunopositivity like the endocervicosis glandular cells, the surface mucinous epithelium lacked HBME-1. Furthermore, on closer examination, while PR was predominantly nuclear in the endocervicosis glandular cells, PR was noted in both the cytoplasm and nuclei of the surface mucinous epithelium; the reasons behind this requiring further elucidation. With this disparity, albeit minor, it is pertinent to consider that the surface mucinous epithelium may or may not be related to the endocervicosis epithelium which it morphologically resembles. That the surface mucinous epithelium is purported secondary mullerian tissue penetrating urothelium in the development of endocervicosis seems quite unlikely in this case. However, the possibility that the surface mucinous epithelium is an implant from the patient's earlier pelvic surgeries, unrelated to the endocervicosis observed, cannot be excluded. It is unfortunate that only the haematoxylin and eosin stained slides of the ovarian mucinous cyst were available for review and paraffin-embedded tissue blocks of resected material could not be obtained for further immunohistochemical investigation. Taking into consideration the similarities and dissimilarities of the surface mucinous and endocervicosis epithelia, incomplete metaplasia of the urothelium while in progression to endocervicosis is another tenable possibility for the surface mucinous epithelium worthy of further deliberation.

**Table 2:** Clinical presentation of bladder lesions with presence of endocervicosis

Author(s) [reference]	Number of cases	Age at presentation (years)	Past medical history	Presentation
Steele and Byrne [1]	1	19	Nil	Urinary tract symptoms Pelvic/abdominal pain
New and Roberts [11]	1	38	1 miscarriage 2 currettages	Urinary tract symptoms
Clement and Young [2]	6	31-44 (mean = 37)	Caesarean sections (2 cases)	Urinary tract symptoms Pelvic/abdominal pain Hematuria Vaginal bleeding Dyspareunia Catamenial exacerbation
Seman and Stewart [12]	1	34	3 caesarean sections	Urinary tract symptoms Pelvic/abdominal pain Catamenial exacerbation
Parivar et al [13]	1	38	Hysterectomy and bilateral salpingo-oophorectomy	Urinary tract symptoms Pelvic/abdominal pain Hematuria
Young and Clement [6]	3	37-46 (mean = 42)	Caesarean section (1 case)	Pelvic mass Pelvic/abdominal pain Incidental finding
Jones et al [14]	1	34	Nil	Urinary tract symptoms
Nazeer et al [15]	6	34-65 (mean = 39)	Hysterectomy (1 case)	Pelvic/abdominal pain Urinary tract symptoms Hematuria Vaginal discharge Incidental finding
Rodriguez and Alfert [16]	1	29	NA	Pelvic/abdominal pain Urinary tract symptoms
Donne et al [8]	1	27	Nil	Urinary tract symptoms Dysmenorrhoea
Spencer et al [17]	1	37	Hysterectomy Salpingo-oophorectomy	Pelvic/abdominal pain Urinary tract symptoms
Kim et al [7]	1	36	NA	Pelvic/abdominal pain Urinary tract symptoms Hematuria
Julie et al [3]	1	35	Uterine curettage	Urinary tract symptoms Catamenial exacerbation
Ozel et al [18]	1	48	Two caesarean sections	Pelvic/abdominal pain Urinary tract symptoms
Campenot et al [19]	1	36	Two caesarean sections	Pelvic/abdominal pain
Koren et al [9]	1	41	Nil	Pelvic/abdominal pain Urinary tract symptoms Hematuria Catamenial exacerbation
Eskridge et al [20]	1	51	Ovarian cystectomy	Pelvic/abdominal pain Urinary tract symptoms Dyspareunia
Joseph et al [21]	1	41	Salpingectomy Caesarean section	Pelvic/abdominal pain Dysmenorrhoea
Preusser et al [22]	1	47	Caesarean section	Incidental finding
Heretis et al [4]	1	67	2 caesarean sections	Flank pain Urinary tract symptoms
Trpkov et al [23]	1	29	NA	Incidental finding
Hao et al [5]	1	34	Caesarean section	Urinary tract symptoms
Guan et al [10]	1	28	Nil	Hematuria

The patient has remained asymptomatic and without evidence of disease recurrence 18-months after transurethral resection of the lesion as would be expected by the currently known natural history of the condition [2,15].

## Conclusions

Endocervicosis in the urinary bladder is a rare but clinically significant entity. The observation in this case, of endocervical-like mucinous epithelium in continuity with urothelium and the slightly differing immunophenotype of this epithelium with that of the endocervicosis glands, is interesting and may provide clues to the pathogenesis of this rare entity.

## Consent

Written informed consent was obtained from the patient for publication of this case report and accompanying images. A copy of the written consent is available for review by the Editor-in-Chief of this journal

## Competing interests

The authors declare that they have no competing interests.

## Authors' contributions

PLC was a major contributor in writing the manuscript. LML, KHT, KSM, ARN participated in the pathological examination of the case. GEGL provided the clinical information and correlation. All authors read and approved the final manuscript.
